# *piggyBac *is an effective tool for functional analysis of the *Plasmodium falciparum *genome

**DOI:** 10.1186/1471-2180-9-83

**Published:** 2009-05-07

**Authors:** Bharath Balu, Chitra Chauhan, Steven P Maher, Douglas A Shoue, Jessica C Kissinger, Malcolm J Fraser, John H Adams

**Affiliations:** 1 Department of Global Health, 3720 Spectrum Blvd Suite 304, University of South Florida, Tampa, Florida 33612, USA; 2 Department of Biological Sciences, University of Notre Dame, Notre Dame, Indiana 46556, USA; 3 Center for Tropical and Emerging Global Diseases, University of Georgia, Athens, Georgia 30602, USA; 4 Department of Genetics, University of Georgia, Athens, Georgia 30602, USA

## Abstract

**Background:**

Much of the *Plasmodium falciparum *genome encodes hypothetical proteins with limited homology to other organisms. A lack of robust tools for genetic manipulation of the parasite limits functional analysis of these hypothetical proteins and other aspects of the *Plasmodium *genome. Transposon mutagenesis has been used widely to identify gene functions in many organisms and would be extremely valuable for functional analysis of the *Plasmodium *genome.

**Results:**

In this study, we investigated the lepidopteran transposon, *piggyBac*, as a molecular genetic tool for functional characterization of the *Plasmodium falciparum *genome. Through multiple transfections, we generated 177 unique *P. falciparum *mutant clones with mostly single *piggyBac *insertions in their genomes. Analysis of *piggyBac *insertion sites revealed random insertions into the *P. falciparum *genome, in regards to gene expression in parasite life cycle stages and functional categories. We further explored the possibility of forward genetic studies in *P. falciparum *with a phenotypic screen for attenuated growth, which identified several parasite genes and pathways critical for intra-erythrocytic development.

**Conclusion:**

Our results clearly demonstrate that *piggyBac *is a novel, indispensable tool for forward functional genomics in *P. falciparum *that will help better understand parasite biology and accelerate drug and vaccine development.

## Background

Malaria is a leading infectious disease that affects 400–600 million people, causing 2–3 million deaths, every year [1]. Out of the four *Plasmodium *species that cause malaria, *Plasmodium falciparum *is responsible for much of the mortality associated with the disease primarily due to lethal infections in young children of sub-Saharan Africa. A continuous rise in parasite drug-resistance has further hindered malaria control strategies and resulted in increased number of deaths in the last few years [2].

The current post-genome era has witnessed a progression of functional genomics studies accomplished in *P. falciparum*, providing valuable information about parasite biology [3-8]. Despite these enormous efforts, *Plasmodium *genomes continue to be perplexing with more than 50% of the genes coding for hypothetical proteins with limited homology to model organisms. High throughput methods for identification of gene functions are imperative to better understand parasite biology and develop effective disease control strategies. However, generating gene disruptions through classic reverse genetic approaches is a complex and inefficient process in *P. falciparum*, due to an extremely low parasite transfection efficiency and the parasite's ability to maintain transfected plasmids as episomes, resulting in only less than 1% of the total annotated genes knocked out thus far [9,10].

Insertional mutagenesis approaches are widely used in prokaryotes and eukaryotes for genome characterizations. Specifically, transposon-mediated mutagenesis has emerged as a powerful molecular genetic tool for eukaryotic transgenesis [11-14] and is extensively used to create gene disruptions, trap promoters and enhancers, and generate gene fusions in model organisms such as *Drosophila *and yeast [12,14]. However, the lack of such advanced genetic approaches in *Plasmodium *is a major impediment to elucidating the parasite genome.

*piggyBac *is a 'cut-and-paste' transposon that inserts into TTAA target sequences in the presence of a *piggyBac *transposase [15,16]. *piggyBac *has gained recent acclamation as a genetic tool due to its functionality in various organisms [17-19] and ability to integrate more randomly into genomes [20]. Moreover, *piggyBac*'s insertion preference for transcription units [17,20] enhances its efficacy in large-scale mutagenesis studies to identify gene functions. We had earlier reported the development of an efficient, *piggyBac*-based system for genetic manipulation of *P. falciparum *[21]. In this study, we improved efficiency of the *piggyBac *transposition system for *P. falciparum *and evaluated its application in whole-genome functional analysis of this most lethal human malaria parasite.

## Results

### Plasmid design, generation of mutant *P. falciparum *clones and insertion site analyses

**Figure 1:**
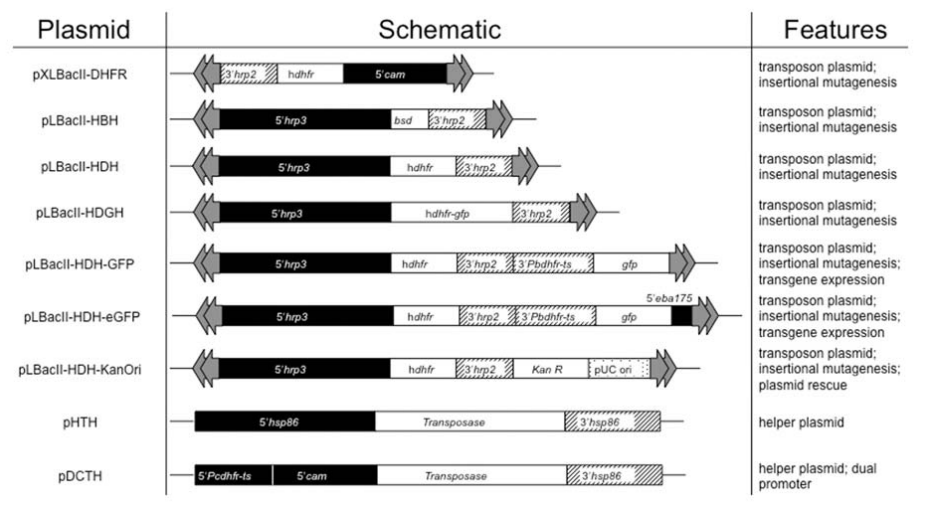
**Plasmid design for *piggyBac *mutagenesis of *P. falciparum***. A summary of different transposon and transposase plasmids tested in *P. falciparum*. Maximum transformation efficiency was obtained while using a dual promoter for transposase expression.

*piggyBac *insertions into the *P. falciparum *genome were obtained by co-transfection of parasite erythrocytic stages with a transposon plasmid and a transposase-expressing helper plasmid as described previously [21]. To optimize the *piggyBac *system for maximum efficiency, several transposon and transposase plasmids were tested in *P. falciparum *(Fig. 1). The transposon plasmids tested contained different regulatory elements and drug selectable markers, which, however, resulted in similar transformation efficiencies (interpreted as the number of *piggyBac *insertions obtained per transfection). As *piggyBac *transposase is the functional enzyme catalyzing the integration event, we hypothesized that increased expression of the transposase with a stronger promoter would result in increased transformation efficiency. The *hsp86 *promoter in the helper plasmid, pHTH [21], was therefore replaced with a previously described dual *Plasmodium *promoter, containing 5' *calmodulin *and 5' *dhfr-ts *regions in head to head orientation [22]. Corroborating our theory, significantly higher transformation efficiencies (an average of 3.1 × 10^-6^) were obtained using the dual promoter for transposase expression as compared to using pHTH (an average of 1.6 × 10^-6^) in approximately 40 transfections each (χ^2 ^test, df 1, P = 0.015).

**Figure 2:**
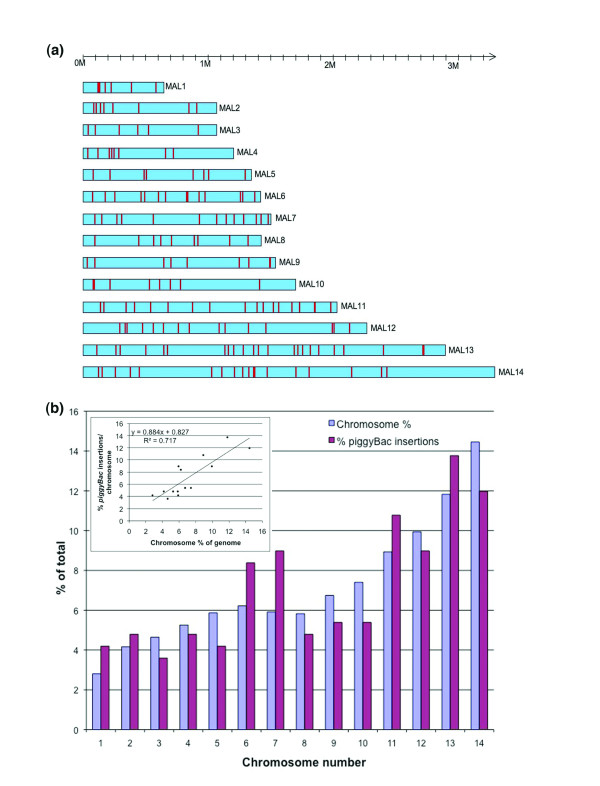
**Distribution of *piggyBac *insertion sites in the *P. falciparum *genome**. **(a) **A representation of the 14 *P. falciparum *chromosomes with *piggyBac *insertion loci (represented by red vertical lines) shows extensive distribution of *piggyBac *insertions through out the parasite genome. **(b) **Comparison of chromosomal distribution of *piggyBac *insertions to the percent genome content of each chromosome shows unbiased insertions into *P. falciparum *genome. Plot and curve fits of percent *piggyBac *insertions and percent chromosome size are depicted in the inset.

**Figure 3:**
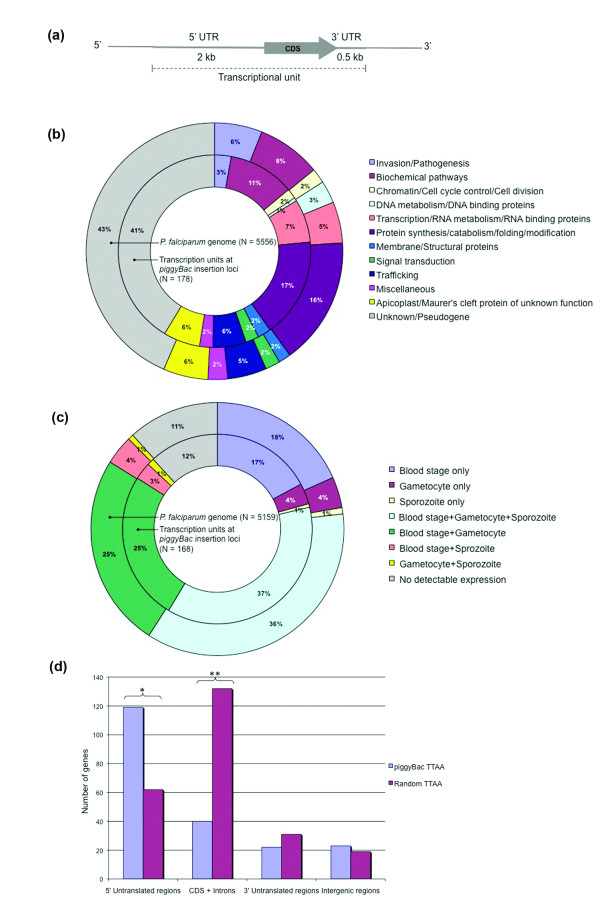
***piggyBac *insertions in the genome are random but preferentially occur in 5' untranslated regions**. (a) Genomic transcription units were defined to include 2 kb of 5' UTR, the coding sequence, the introns and 0.5 kb of 3' UTR, based on previous studies in *Plasmodium *[48,49]. (b) Comparison of gene functions of all annotated genes in the genome (outer circle) to genes in *piggyBac*-inserted loci (inner circle) shows an equivalent distribution confirming random insertions in the parasite genome. (c) Comparison of stage-specific expression of all annotated genes (outer circle) to those in *piggyBac*-inserted loci (inner circle) validates the ability of *piggyBac *to insert in genes expressed in all parasite life cycle stages. (d) A comparison of *piggyBac*-inserted TTAA sequences to TTAA sequences randomly selected from the genome showed preferential insertion of *piggyBac *into 5' UTRs of genes (asterisk- χ^2 ^test, df 1, P = 1.5 × 10^-12^) whereas a significantly lower number of insertions were observed in CDS and introns (double asterisks- χ^2 ^test, df 1, P = 1.09 × 10^-13^).

Following transfection with *piggyBac *plasmids, drug resistant parasite populations were established rapidly, within 2–3 weeks and the total number of *piggyBac *insertions obtained per transfected parasite population varied from 1 to 14. Through 81 independent transfections, we generated 177 unique mutant clones of *P. falciparum *with *piggyBac *insertions in their genomes. Southern blot hybridization analysis of parasite clones, derived by limiting dilution of drug-resistant populations, revealed single *piggyBac *insertions in all except two clones that had two insertions each (data not shown). Also, none of the mutant clones retained the *piggyBac *plasmid as episomes indicating highly efficient transposition events (data not shown). Out of the 179 *piggyBac *insertions identified, 165 could be mapped unambiguously on the *P. falciparum *genome by performing BLAST searches using NCBI http://www.ncbi.nlm.nih.gov/genome/seq/BlastGen/BlastGen.cgi?taxid=5833 and PlasmoDB [23] databases. The remaining 14 insertions either mapped to telomeric repetitive elements or could not be mapped to a chromosomal location through BLAST searches of public databases. The identified *piggyBac *insertion sites were distributed throughout the genome in all 14 *P. falciparum *chromosomes (Fig. 2a) with no bias for any particular chromosome (Fig. 2b). All *piggyBac *insertions were obtained in the expected TTAA target sequences except two that integrated into TTAT and TTAG sequences. As in other organisms [17,20], *piggyBac *preferentially inserted into predicted transcribed units of *P. falciparum *genome (Fig. 3a), affecting 178 transcription units. Thirty-six of the insertions resulted in direct disruption of open reading frames (ORFs) and 3 insertions were mapped to introns. A vast majority of insertions (119) occurred in 5' untranslated regions (UTRs) whereas only a few (22) were obtained in 3' UTRs (Additional file 1).

### *piggyBac *inserts randomly into all categories of genes with a strong preference for 5' untranslated regions

Obtaining unbiased insertions into the genome is critical for whole-genome mutagenesis and other large-scale analyses. Hence, we evaluated the randomness of *piggyBac *insertions into the *P. falciparum *genome by comparing the functional categories of genes with *piggyBac *insertions to all annotated genes in the genome. An identical functional distribution of genes was seen in both *piggyBac *insertion loci and the genome (Fig. 3b) except for fewer insertions in genes involved in DNA metabolism/DNA-binding and invasion/pathogenesis (Fisher's exact test, P = 0.038 and P = 0.04, respectively). Since the parasite erythrocytic stages were used for *piggyBac *transformation, we further investigated the bias for *piggyBac *insertions in erythrocytic stage genes relative to genes expressed in other stages of development. By utilizing the gene expression profiling data for *P. falciparum *[3], we classified all annotated genes based on their expression in different parasite life cycle stages and confirmed unbiased *piggyBac *insertions in genes expressed in all parasite stages (Fig. 3c). A separate comparison of genes with *piggyBac *insertions in coding sequences only to all genes also revealed no significant insertion bias for any functional category or stage of expression (data not shown).

Even though transposon-mediated mutagenesis is a relatively random process, preferential insertion into genomic hotspots is characteristic of some transposons [20]. In our studies, we observed a significantly higher number of *piggyBac *insertions in 5' UTRs and a significantly lower number in coding sequences, relative to a distribution of 214 randomly selected genomic TTAA sequences (Fig. 3d).

### A putative motif for *piggyBac *insertion in the *P. falciparum *genome

**Figure 4:**
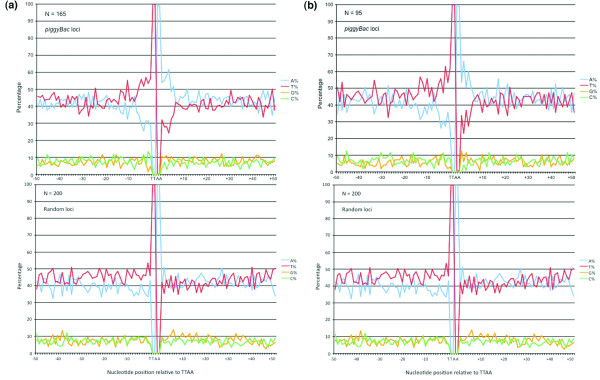
***piggyBac *inserts into AT-rich regions of the *P. falciparum *genome**. (a) Nucleotide composition analysis of the flanking sequences showed that *piggyBac *inserted TTAA sites preferentially occur in the middle of an AT-rich core of 10 nucleotides predominantly with 'T's upstream (χ^2 ^test, df 1, P = 6.3 × 10^-5^) and 'A's downstream (χ^2 ^test, df 1, P = 2.07 × 10^-8^) as compared to randomly selected genomic TTAA sequences. (b) A comparison of nucleotide composition of flanking sequences only in the 5' untranslated regions (UTRs) of *piggyBac *inserted and randomly selected TTAA sequences further confirms the specificity of *piggyBac *for AT-rich target sites.

Previous studies in other organisms had observed some AT-richness around *piggyBac *insertion sites [17,24]. However, it was somewhat surprising that our analysis of a 100 bp flanking region showed a significantly higher AT-content around *piggyBac *inserted TTAA sequences (average AT content of 85.56%) as compared to random TTAA sequences (average AT content of 80.24%), in the already AT-rich *P. falciparum *genome (two-tailed t-test, P = 2.95 × 10^-13^). A closer look at the *piggyBac *insertion sites revealed their presence in the middle of an AT-rich core of 10 nucleotides predominantly with 'T's upstream and 'A's downstream (Fig. 4a, upper panel). No such signature motif was present around the randomly selected TTAA sequences either from the genome (Fig. 4a, lower panel). Even when only analyzing the genomic 5' UTRs, a similar bias in the insertion site selection existed (Fig. 4b).

### Validation of a phenotypic screen in *P. falciparum*

**Figure 5:**
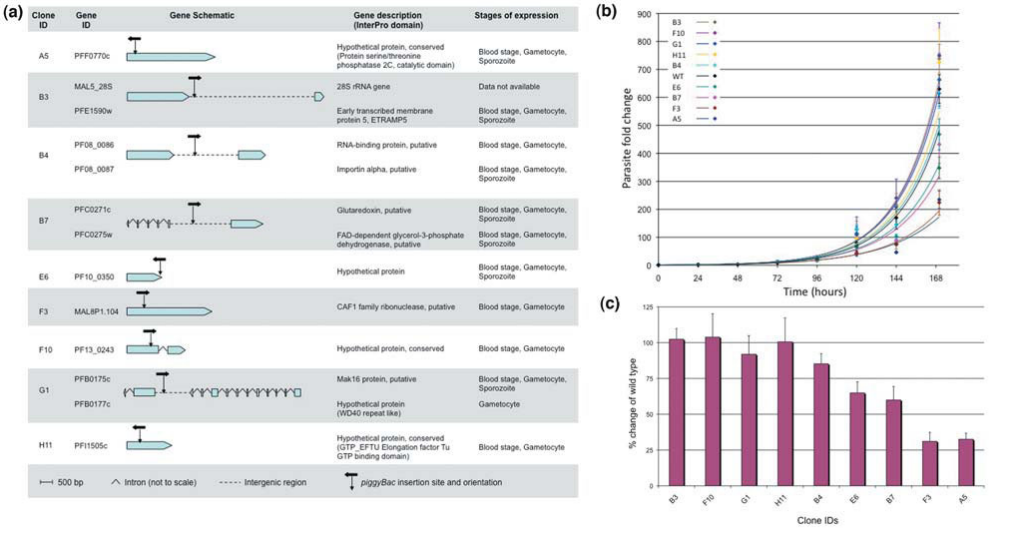
**A phenotype screen for attenuated blood-stage growth**. (a) A schematic of mutant *P. falciparum *clones selected for growth rate analysis. Black vertical and horizontal arrows indicate the insertion site and orientation of the *piggyBac *transposon, respectively. The gene schematic, description and expression stages were all obtained from the PlasmoDB database at http://www.plasmodb.org. (b) Growth curves of 9 insertional mutant clones, were obtained by plotting parasite fold change against time. For the wild type (WT), an average of fold changes from three different NF54 clones was used. The order of samples, from top to bottom, indicates a decrease in parasite fold changes. (c) A bar-graph of fold changes in parasite numbers after 7 days of growth revealed a spectrum of attenuated growth phenotypes in several mutant clones when compared to the wild type clones. The error bars in (b) and (c) represent standard deviation from the mean of 3 measurements.

The preferential insertion of *piggyBac *into transcription units prompted us to investigate the feasibility of forward genetic studies in *P. falciparum *that have been completely lacking thus far. Little is known about what metabolic pathways and processes are essential for parasite growth and survival in the blood of the vertebrate host, and therefore we screened the erythrocytic stages of *P. falciparum *mutant clones for attenuated growth phenotypes. We first screened for mutant clones that appeared to have aberrant growth rate by standard light microscopy methods and then studied them further by performing more precise growth assays. The mutant clones selected for growth analysis contained single *piggyBac *insertions in their genomes in either coding sequences or 5' UTRs and were associated with several metabolic pathways (Fig. 5a). To confirm that *piggyBac *insertion into the genome alone does not affect growth, additional mutant clones were included as controls. An exponential growth curve was generated for each mutant clone by estimating parasitemias every 24 hrs for 7 days using flow cytometry as described before [25,26] with some modifications. Four mutant clones (A5, B7, E6 and F3) displayed significantly reduced growth rate as compared to five other insertional mutants (B3, B4, F10, G1, and H11) and the wild type (WT) clones (Fig. 5b). The experiment was performed three times, with two sub-clones for each mutant and similar results were obtained in all experiments (data not shown). The parasite exponential growth curve was further used to estimate the individual doubling times of the mutant clones as described previously [26] that confirmed the observed attenuated phenotypes (Table 1, See Fig. S1 in Additional file 2). Knock out of gene expression was confirmed in clones with insertions in coding sequences by RT-PCR (See Fig. S2 in Additional file 3). Clones A5 and F3 with insertions in the coding sequences of PFF0770c and MAL8p1.104, respectively, were the most affected with an approximate growth rate of only 30% as compared to the WT clones (Fig. 5c). The attenuated growth rates observed in these mutant clones substantiate their significance in intra-erythrocytic development of the parasite, though additional studies are required to characterize the attenuation mechanisms.

**Table 1:** Doubling time estimation of *P. falciparum *mutant clones

Clone ID	Doubling time estimate (hours)	Standard error	95% CI		P value	t value	df
A5	22.07	0.26	21.53	22.60	**0.00007**	7.4656	7
B3	17.89	0.06	17.77	18.00	0.97376	-2.3316	7
B4	18.45	0.10	18.25	18.66	0.41380	0.2261	7
B7	19.70	0.17	19.34	20.06	**0.00368**	3.7297	7
E6	19.28	0.12	19.04	19.52	**0.00565**	3.4086	7
F3	21.98	0.17	21.64	22.33	**0.00001**	10.5459	7
F10	17.83	0.09	17.64	18.03	0.97735	-2.4318	7
G1	18.17	0.08	18.02	18.33	0.83353	-1.0400	7
H11	18.03	0.11	17.80	18.26	0.89928	-1.4098	7
WT	18.39	0.06	18.26	18.52	N/A: WT is reference		

Doubling times for mutant parasite clones were estimated using their respective best-fit growth curves (See Fig. S1 in Additional file 2). Simple two-tailed t-test was then used to test the significance of differences in doubling time of mutant clones with wild type (WT) *P. falciparum *clones (average of three NF54 clones) as the reference. Significant P values, based on alpha = 0.05, are highlighted in bold.

## Discussion

Persistent problems with drug resistance and the critical need to identify novel targets for therapeutic intervention creates a continuing need to improve our understanding of what is important for growth and development of malaria parasites. A major barrier in experimental malaria research has been a limited ability to manipulate *P. falciparum *genes to determine their functions and associated pathways of interactions within the parasite. Large-scale mutagenesis screens are vital for improving our understanding of *Plasmodium *biology and functional analysis of its genome. Random transposon mutagenesis is a powerful approach to identify critical biological processes in an organism and is an approach successfully applied in numerous eukaryotes [11-13]. In particular, *piggyBac *has become widely used to manipulate genomes and is currently the preferred vector of choice for gene discovery and validation of gene function in *Drosophila *and the laboratory mouse [17,20,27-30]. We therefore evaluated *piggyBac *as a novel genetic tool for the functional analysis of the *P. falciparum *genome.

Several transposon and transposase plasmids were created and tested in *P. falciparum *for maximum transformation efficiency. All the plasmids tested transformed with similar efficiencies except for the helper plasmid, pDCTH, with the double promoter that almost doubled the transformation efficiency. There were no apparent differences in integration specificities of the various plasmids as insertions in the genome were randomly distributed in all cases. The presence of different selectable markers, hDHFR and BSD, in plasmids pLBacII-HDH and pLBacII-HBH respectively, allows re-transformation and complementation of previously transformed drug-resistant *P. falciparum *clones. The plasmids pLBacII-HDH-GFP and pLBacII-HDH-eGFP can trap promoters in the genome if inserted in the right orientation downstream to an endogenous promoter as shown previously [31]. These plasmids can also be modified for stable transgene expression with or without GFP tag. Parasites transformed with pLBacII-HDGH, with hDHFR-GFP fusion as selectable marker, display high levels of fluorescence and are amenable to sorting by Fluorescence activated cell sorter (FACS). Transformation with the plasmid pLBacII-HDH-KanOri inserts the kanamycin resistance gene and a pUC origin of replication into the parasite genome that allows for plasmid rescue from the genome for easy identification of insertion sites.

The genome-wide integration of *piggyBac *into genes in all functional categories, expressed in all parasite life cycle stages, validates its application in whole-genome mutagenesis of *P. falciparum*. Almost all mutant *P. falciparum *clones generated had single *piggyBac *insertions in their genomes, which will aid in easy correlation of mutant phenotypes to their respective genotypes. The increased number of insertions obtained in 5' UTRs of genes indicates either active changes in chromatin structure allow easy access for *piggyBac *to the genomic DNA or the affinity of the transposase for chromatin associated factors unique to these regions. Alternatively, this skewed distribution could simply be the inability to recover mutants with insertions in coding sequences of essential genes, whereas insertions in 5' UTRs of essential genes may not completely abolish gene expression and hence may not be lethal.

From whole-genome mutagenesis perspectives, insertions in 5' UTRs may have a varied effect on neighbouring gene expression. Insertions in 5' UTRs could either increase gene expression, possibly due to better recruitment of transcription machinery, or decrease gene expression by blocking transcription. A meaningful approach would therefore be to subject all 5' UTR mutants to phenotypic analyses as either increased or decreased gene expression can significantly alter intracellular activities. Such a scenario might be particularly beneficial in identifying essential genes that cannot be knocked out in the parasite. Nevertheless, 22% of the insertions were obtained in coding sequences generating 39 gene knockouts, which almost equal the number of unique gene knockouts generated in *P. falciparum *thus far until a recent large-scale study achieving 53 gene knockouts [32], using conventional methods [10]. Such high propensity to create gene disruptions and the ability to rapidly generate stable lines of mutant clones, warrants the use of *piggyBac *in large-scale mutagenesis studies not only to identify gene functions, but also to discriminate the essential and dispensable regions of the parasite genome that will further confine the search for potent drug targets.

The most significant application of random mutagenesis is the ability to perform forward genetic screens to select mutants of a desired phenotype. Our limited phenotypic screen for attenuated parasite growth confirmed the feasibility of such approaches in *P. falciparum *by identifying several genes and pathways critical for blood-stage development. One of the most severely affected mutant parasites identified in our screen is a knockout of MAL8P1.104 (clone F3), which is the *Plasmodium *orthologue of yeast *Caf1 *(*CCR4-associated factor 1*) [33]. In yeast, CAF1 is a component of CCR4-NOT complex that is a global regulator of gene expression, controlling chromatin remodelling, transcriptional regulation, mRNA stability and protein degradation [34]. Experimental protein interaction data indicates a similar functional complex exists in *P. falciparum *[7] and with a scarcity of known transcription factors or identifiable conserved regulatory elements in *Plasmodium*, deadenylation may be extremely significant in controlling gene expression through regulating mRNA abundance by degradation [35].

The significance of protein phosphorylation and dephosphorylation in regulating parasite cellular activities is also clearly demonstrated by the attenuated growth phenotype of our knockout of PFF0770c (clone A5), which encodes one of the 12 type 2C protein phosphatases (PP2C) found in *Plasmodium *[36]. PP2Cs carry out a wide range of functions in higher eukaryotes including intracellular signalling and providing cell cycle and developmental check points [37-39]. Two PP2Cs, in the closely related apicomplexan *Toxoplasma*, were recently shown to be involved in parasite motility and host cell modulation [40,41].

Another mutant clone displaying attenuated growth was a knockout of PF10_0350 (clone E6) that codes for a hypothetical protein unique to *Plasmodium *species and attests to the theory that such unique *Plasmodium *genes need to be investigated further as antimalarial targets. *piggyBac *insertion in the 5' UTRs of PFC0271c and PFC0275w, coding for glutaredoxin and glycerol-3 phosphate dehydrogenase, respectively, resulted in increased levels of both transcripts in the mutant clone B7 as seen by quantitative RT-PCR (data not shown), indicating that optimal expression of genes is essential for normal parasite growth.

Several other phenotypic screens such as those for virulence, drug resistance, gametocytogenesis and transmissibility of infection to mosquito hosts can now be accomplished in *P. falciparum *that will contribute immensely to our current understanding of parasite biology.

Apart from its application in whole-genome mutagenesis and phenotype screens, *piggyBac *is also a powerful tool for stable transgene expression in *P. falciparum *as any parasite strain or clone of interest can be transformed. We have confirmed the functionality of *piggyBac *system in three different strains of *P. falciparum *thus far, including, NF54, 3D7, and HB3, and have obtained genomic insertions of up to 7 kb of DNA (including the drug selection cassette) with no reduction in transformation efficiency (Balu and Adams, unpublished data) [42]. The ability to express transgenes stably from the genome offers numerous possibilities to study various biological aspects of the parasite such as, coordinated gene expression, phenotypic effects of copy number variations and protein trafficking.

## Conclusion

Despite years of efforts, *Plasmodium *biology remains puzzling due to its complexity and refractoriness to routine genetic analyses. By using the *piggyBac *transposable element in *P. falciparum*, we have clearly demonstrated the possibility of whole-genome mutagenesis and forward functional genomics in this lethal malaria parasite that will drastically advance our understanding of *Plasmodium*'s parasitic and pathogenic abilities and quicken the search for new drug targets and vaccine candidates.

## Methods

### Plasmid constructs

*piggyBac *plasmids used for transfections were derived from previously reported plasmids pXL-BACII-DHFR and pHTH [21].

pLBacII-HDH-pXL-BacII-DHFR was digested with XhoI and the site was removed by filling in the overhangs with klenow and religation to yield pLBacII-DHFR. The human DHFR selection cassette in pLBacII-DHFR was then replaced with a different human DHFR drug selection cassette from the plasmid pHD22Y [43] using EcoRI/BamHI to yield pLBacII-HDH.

pLBacII-HDH-GFP- The *gfp *coding sequence along with 3' *Pbdhfr *was amplified as a single fragment from the vector pHH2 [44] by PCR with extensions for restriction sites SpeI and ApaI using primers F-*ACTAGT*GCGGCCGCCTACCCT and R-*GGGCCCGGTACC*CTCGAGATCTTAGAATGAAGATCTTATTAC. The PCR product was then cloned into pGEM-Teasy vector (Promega) and sub-cloned into pLBacII-HDH using ApaI and SpeI.

pLBacII-HDH-eGFP- A 200 bp region of 5' *eba-175 *was amplified from the *P. falciparum *genome using primers F-*ATCGAT*GAATATAATTGATTGATTGTAATAAAAAGTG and R-*GGGCCC*TGTATGCACATTGAATATATTTATATGTTATTATC and cloned into pLBacII-HDH-GFP as a ClaI/ApaI fragment.

pLBacII-HDH-KanOri- The kanamycin resistance gene and pUC origin of replication were amplified as a single fragment by PCR from the vector pEGFP-C1 (Clontech) using primers F-ATGATGATG*GGATCC*AAATGTGCGCGGAACCCC and R-ATGATGATG*GGATCC*GCAAAAGGCCAGCAAAAGG and cloned into pGEM-Teasy vector (Promega). The fragment was then sub-cloned into the plasmid pLBacII-HDH as a BamHI fragment.

pLBacII-HBH- The hDHFR coding sequence was first cut out from the vector pHD22Y using NsiI and HindIII and replaced with the blasticidin-S-deaminase (BSD) coding sequence that was cut out from the vector pCBM-BSD [45] using NsiI and HindIII. The BSD selection cassette in pHD22Y was then moved as an EcoRI/BamHI fragment into the vector pL-BacII-DHFR to yield pLBacII-HBH.

pLBacII-HDGH- The hDHFR-GFP fusion gene was cut out from the vector pHDGFP2 [46] using NsiI and HindIII and cloned into pHD22Y replacing the human DHFR coding sequence. The whole selection cassette was then moved as an EcoRI/BamHI fragment into the vector pLBacII-DHFR to yield pLBacII-HDGH.

pDCTH- The plasmid with a dual promoter for transposase expression was created by PCR amplifying 5' *Pcdhfr-ts *and 5' *calmodulin *as an EcoRI fragment from the plasmid pHC1-CAT [22] using primers F-ATGATG*GAATTC*CCTGATATATTTCTATTAGGTATTTATTA; R-ATGATG*GAATTC*TTTGTAAGTTTTAGGTGTGTGTAT and swapping it with the 5' *hsp86 *region in the helper plasmid, pHTH.

### Parasite culture and transfection

*P. falciparum *clone NF54 was cultured in human erythrocytes at 5% hematocrit in RPMI1640 medium containing 0.5% Albumax II, 0.25% sodium bicarbonate and 0.01 mg/ml gentamicin. Transfections were performed using red blood cells as described previously [21]. Briefly, mature blood-stage parasites were purified on a MACS magnetic column (Miltenyi Biotec) and 1 million purified parasites were added to erythrocytes loaded with 100 μg of the transposon plasmid and 50 μg of the transposase plasmid to start a 5 ml parasite culture. Individual mutant clones were obtained by limiting dilution of parasites post-drug selection.

### Identification of *piggyBac *insertion sites

Genomic DNA (2 μg) extracted from transformed parasites was digested with 10 units of either Dra I or Rsa I and used either in inverse PCR [21] or vectorette PCR reactions according to manufacturer's instructions (UVS1 Vectorette™ Genomic Systems, Sigma). The amplified PCR products were sequenced with primers in *piggyBac *inverted terminal repeats [21] and analyzed using MACVECTOR software (MacVector, Inc.). Insertion sites were identified by performing BLAST searches using NCBI http://www.ncbi.nlm.nih.gov/genome/seq/BlastGen/BlastGen.cgi?taxid=5833 and PlasmoDB databases [23].

### Parasite growth assays, flowcytometry and estimation of doubling times

Growth assays were performed by maintaining asynchronous cultures of *P. falciparum *wild type and mutant clones at parasitemias 0.5–2% in 96-well plates by diluting every 48 hrs. Parasite cultures were plated in triplicates for each time point and samples were taken every 24 hrs for 7 days and fixed in 0.05% glutaraldehyde after removal of culture medium. Flow cytometry was used to estimate parasitemia as described before [25,47] by staining parasites with Ethidium bromide and analyzing using FACSCanto™ II flowcytometry system (Becton, Dickinson and Company) in a high throughput format. A total of 20,000 cells were counted for each sample. The data were analyzed using FACSDIVA™ software (Becton, Dickinson and Company). Growth rate (defined as the change in parasite numbers every 24 hrs over a period of 7 days) analyses were performed using SAS (9.1). The total number of parasites (y) (parasitemia × dilution factor), was plotted against time (×) and fitted to the exponential growth curve

where, D is the intrinsic parasite doubling time and m0 is the theoretical parasite number at time 0. To compare directly the growth rate of parasite clones with slightly different starting parasitemias, the -fold increase of the parasite number, normalized to have a single theoretical parasite for each culture at time 0, was used for graphing the growth curve [26]. One hundred parameter initiation values ranging from 5 to 105 were tested and the best converging model with the smallest Sum Square of Error (SSE) was chosen for estimation of doubling time.

## Authors' contributions

BB, SM and DAS performed the transfections. BB, CC and SM performed the growth rate experiments. BB, CC, JCK, and JHA analyzed the insertions data. BB, CC, SM and JHA analyzed the growth rate data. CC, JCK and MJF contributed reagents/materials/analysis tools. BB, CC and JHA drafted the manuscript. BB, MJF and JHA conceived and designed the study. All authors read and approved the final manuscript.

## Supplementary Material

Additional file 1 **List of *piggyBac *insertion loci in the *P. falciparum *genome**. Complete list of *piggyBac *insertion loci identified thus far is provided along with the mutant name and insertion position relative to the coding sequences of the genome. Click here for file

Additional file 2 **Best-fit growth curve models for doubling time estimation of mutant clones**. The predicted best-fit and observed growth curves for each parasite clone is shown. Click here for file

Additional file 3 **Lack of gene expression in mutant *P. falciparum *clones with insertions in the coding sequences**. RT-PCR analysis confirms the knockout of gene expression in mutant clones, selected for growth assays, with insertions in coding sequences. Click here for file
