# Supplementary material for: piggyBac is an effective tool for functional analysis of the Plasmodium falciparum genome
Source: BMC Microbiol. 2009 May 7;9:83. doi: 10.1186/1471-2180-9-83 (PMC2686711; doi:10.1186/1471-2180-9-83)
Supplement: Additional file 2 — Best-fit growth curve models for doubling time estimation of mutant clones. The predicted best-fit and observed growth curves for each parasite clone is shown. [file 1471-2180-9-83-S2.pdf]

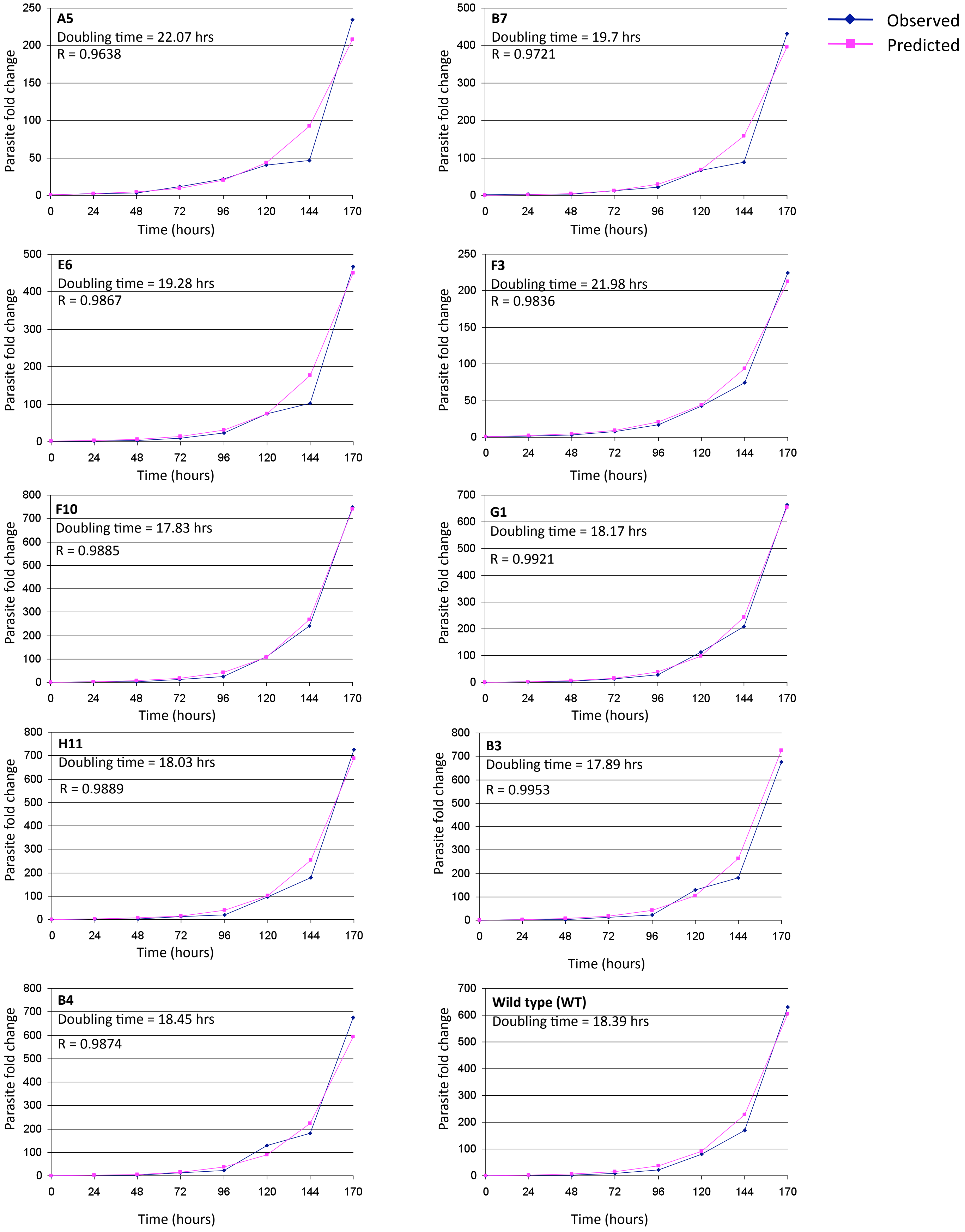

Figure S1: Best-fit growth curve model for doubling time estimation of mutant clones. The predicted best-fit and observed growth curves for each parasite clone is shown.
