# Supplementary material for: piggyBac is an effective tool for functional analysis of the Plasmodium falciparum genome
Source: BMC Microbiol. 2009 May 7;9:83. doi: 10.1186/1471-2180-9-83 (PMC2686711; doi:10.1186/1471-2180-9-83)
Supplement: Additional file 3 — Lack of gene expression in mutant P. falciparum clones with insertions in the coding sequences. RT-PCR analysis confirms the knockout of gene expression in mutant clones, selected for growth assays, with insertions in coding sequences. [file 1471-2180-9-83-S3.pdf]

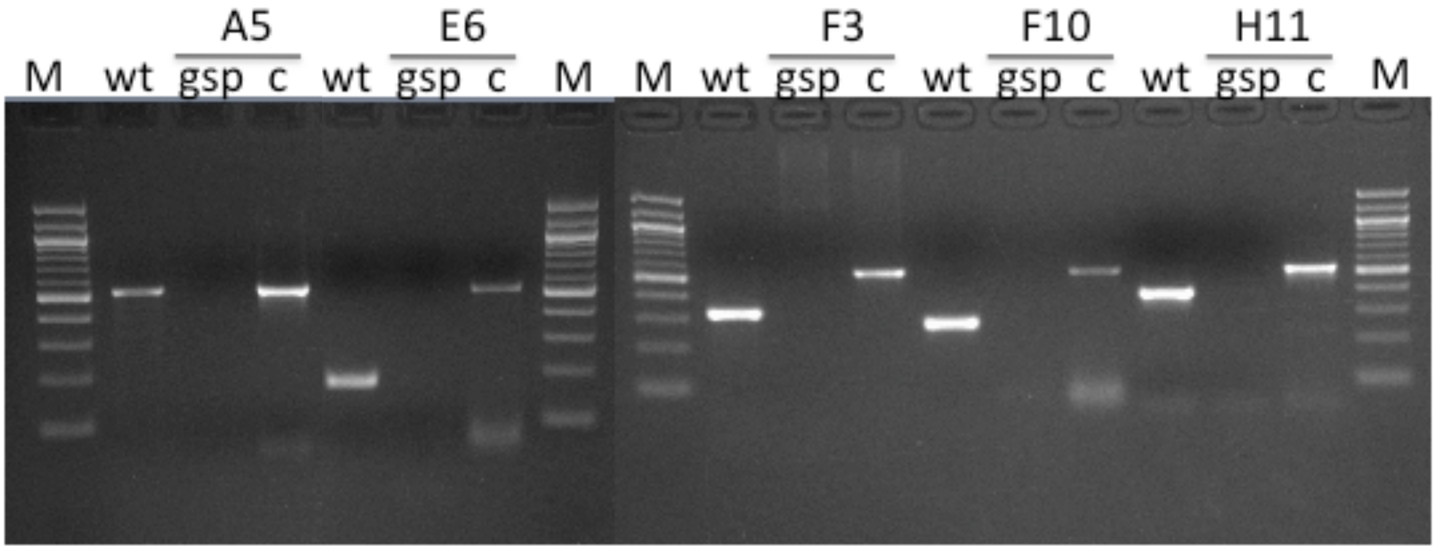

M- Marker
wt- wild type NF54 with respective gene specific primer
gsp- gene specific primers
c- control primers (5′ *maebl*)

Figure S2. Confirmation of lack of gene expression in mutant *P. falciparum* clones selected for growth assays. RT-PCR with primers flanking the *piggyBac* insertion sites confirmed the lack of gene expression in mutant clones with insertions in coding sequences.
